# In Memoriam: George Atkinson Pankey, MD, MACP, FIDSA

**DOI:** 10.31486/toj.26.5063

**Published:** 2026

**Authors:** Ronald G. Amedee, Joseph R. Dalovisio

**Affiliations:** ^1^ Director of Clinical School and Professor, The University of Queensland Medical School, Ochsner Clinical School, New Orleans, LA; Editor-in-Chief, *Ochsner Journal*; ^2^ Department of Philanthropy, Ochsner Clinic Foundation, New Orleans, LA

**Figure:**
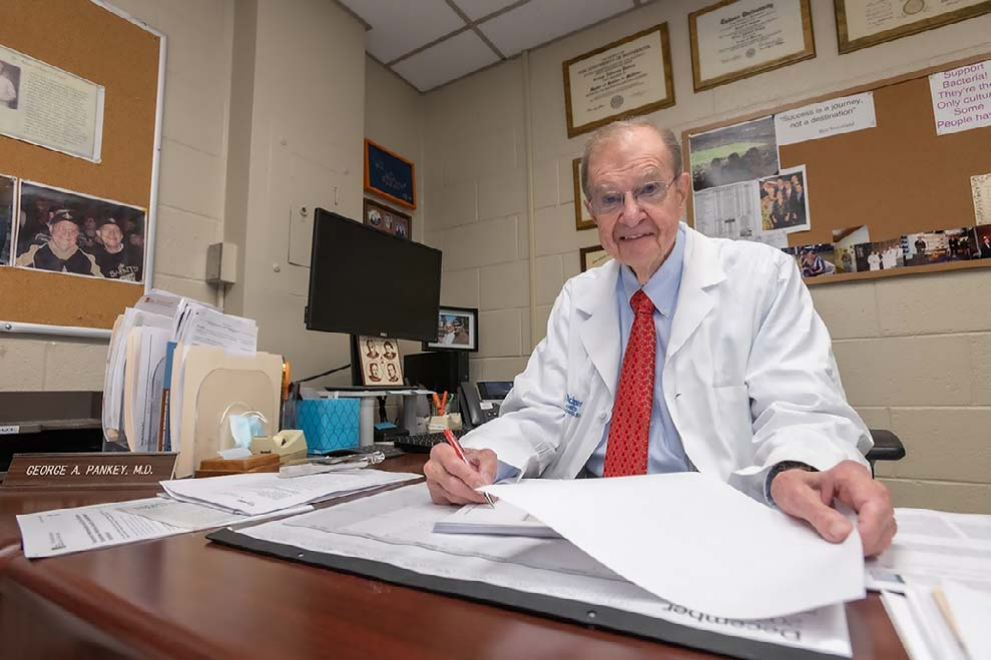
**George Atkinson Pankey, MD, MACP, FIDSA 1933 – 2026**

Dr George Atkinson Pankey was an infectious disease (ID) specialist before ID was even a specialty.

In 1963, he was hired as a consultant at Ochsner Clinic, established a new ID “department,” and functioned as a “department” of one for almost a decade.

In the early 1970s, when the American Board of Internal Medicine introduced the new medical specialty of ID, Dr Pankey took the first examination and was one of the first physicians in the country to achieve board certification. Dr Pankey's colleagues at Ochsner say he may have even contributed questions to that initial exam.

In 1972, ID officially obtained departmental status at Ochsner, and Dr Pankey recruited faculty to support the growing specialty. During his 6 decades at Ochsner, Dr Pankey chaired the department, supervised its growth, and contributed many advancements in ID knowledge via clinical trials and medical research. He also found time to educate interns, residents, and ID fellows. He founded the Ochsner ID fellowship and served as its program director. Several of the more than 50 fellows who trained with Dr Pankey decided to remain on the Ochsner staff after completing their training.

In 1999, he established the ID Translational Research Laboratory. His research focused on novel antibiotic agents and combinations of antibiotics for treating multidrug-resistant bacteria and fungi and the evaluation of rapid diagnostic methods for ID. Dr Pankey had a passion for teaching and often encouraged ID fellows, residents, and medical students to join his research lab and work on a study. In return, he mentored each of them, shared ideas, gave advice, and always assisted with the preparation of a scientific presentation or a manuscript for publication. In addition, Dr Pankey never hesitated to offer a letter of recommendation.

**Figure:**
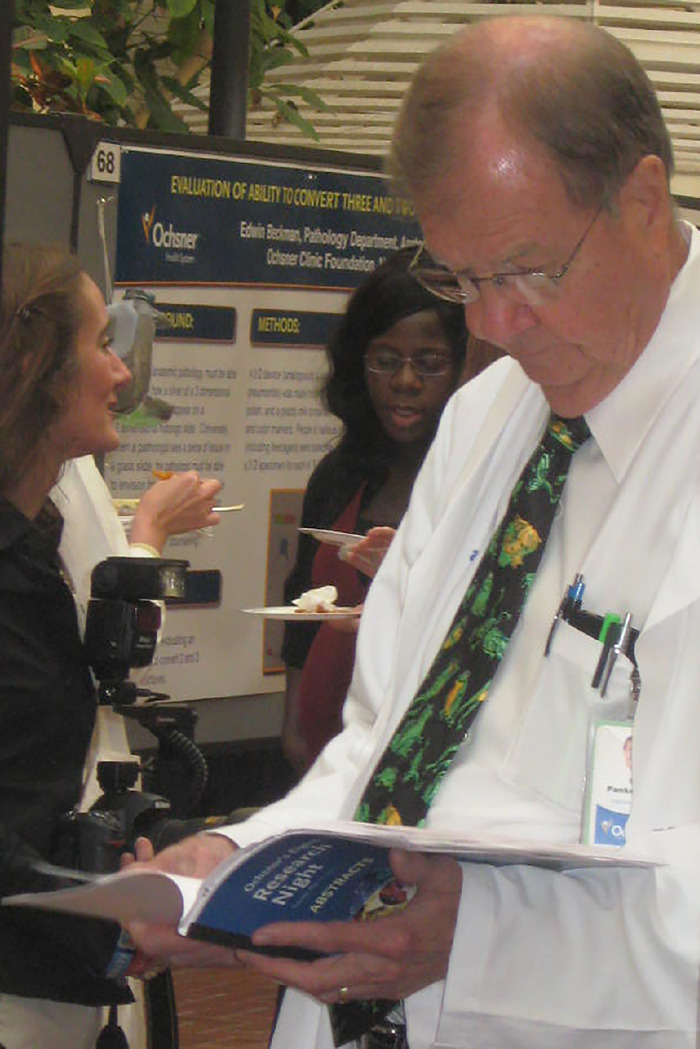
**Dr Pankey reads the program book at Research Night 2011.**

He loved inviting the ID research team (clinical and translational) involved in a study to have regular lunch meetings, where he often provided food for everyone, enjoyed personal chats, and inquired how each person was doing.

He published prolifically, authoring 7 books, 37 book chapters, and 172 peer-reviewed publications. One of his last peer-reviewed articles was published in the Spring 2025 issue of *Ochsner Journal*: “Evaluation of the Diagnostic Accuracy of the T2Resistance Panel (Research Use Only) in Patients With Possible Bacterial Bloodstream Infections.” Dr Pankey was such a stickler for accuracy that he insisted on publishing an erratum after the paper was published to correct 3 minor errors.

Dr Pankey was a member of numerous medical societies and received multiple regional, national, and international awards and honors. He was a highly accomplished teacher, researcher, and clinician and was a role model for trainees and colleagues at every level of the academic learning continuum.

However, he was best known for the high-quality and delicate touch he extended in caring for his challenging patients, many of whom today can gratefully say, “Dr Pankey saved my life.” He demonstrated daily what it means to live a medical career and exhibit the highest level of commitment to the Ochsner Health mission, vision, and values.

Dr Pankey was also enthusiastic about tasty food, fine wines and spirits, and meaningful traditions. He often ended meals with his professional colleagues by toasting with Strega (an Italian liqueur made from 70 secret herbs), lifting his glass to honor their enduring friendship and the lives they had saved.

Dr Pankey, known to many by his nickname “Kin,” was 92 years old when he died on March 31, 2026. He will be deeply missed by his patients, friends, family members, and professional colleagues whose lives he enriched at Ochsner and elsewhere.

